# Efficient and modular reverse genetics system for rapid generation of recombinant severe acute respiratory syndrome coronavirus 2

**DOI:** 10.71150/jm.2504015

**Published:** 2025-07-21

**Authors:** Sojung Bae, Jinjong Myoung

**Affiliations:** Korea Zoonosis Research Institute, Department of Bioactive Material Science and Genetic Engineering Research Institute, Jeonbuk National University, Jeonju 54531, Republic of Korea

**Keywords:** SARS-CoV-2, reverse genetics, cDNA, recombinant virus

## Abstract

The global spread of COVID-19 has underscored the urgent need for advanced tools to study emerging coronaviruses. Reverse genetics systems have become indispensable for dissecting viral gene functions, developing live-attenuated vaccine candidates, and identifying antiviral targets. In this study, we describe a robust and efficient reverse genetics platform for severe acute respiratory syndrome coronavirus 2 (SARS-CoV-2). The system is based on the assembly of a full-length infectious cDNA clone from seven overlapping fragments, each flanked by homologous sequences to facilitate seamless assembly using the Gibson assembly method. Individual cloning of each fragment into plasmids enables modular manipulation of the viral genome, allowing rapid site-directed mutagenesis by fragment exchange. Infectious recombinant virus was successfully recovered from the assembled cDNA, exhibiting uniform plaque morphology and genetic homogeneity compared to clinical isolates. Additionally, fluorescent reporter viruses were generated to enable real-time visualization of infection, and the effects of different mammalian promoters on viral rescue were evaluated. This reverse genetics platform enables efficient generation and manipulation of recombinant SARS-CoV-2, providing a valuable resource for virological research and the development of preventive and therapeutic antiviral measures.

## Introduction

The emergence of SARS-CoV-2 in late 2019 precipitated a global pandemic of unprecedented scale, fundamentally altering public health practices, economies, and societal norms (Jamison et al., 2022; Myoung 2022; Raharinirina et al., 2025; Sachs et al., 2022). This novel coronavirus, belonging to the Betacoronavirus genus, shares significant sequence homology with SARS-CoV, the causative agent of the 2002–2003 SARS outbreak, and exhibits a zoonotic origin likely linked to bats (Worobey et al., 2022). Initial cases identified in Wuhan, China, rapidly spread across international borders, driven by the virus's efficient human-to-human transmission through respiratory droplets and aerosols (Althouse et al., 2020). Characterized by a wide spectrum of clinical manifestations, SARS-CoV-2 infection, termed COVID-19, ranges from asymptomatic carriage to severe pneumonia, acute respiratory distress syndrome (ARDS), multi-organ failure, and death (Acosta et al., 2021). The early phase of the pandemic was marked by high case fatality rates, particularly among elderly individuals and those with underlying health conditions, overwhelming healthcare systems worldwide (Stokes et al., 2021).

Virologically, SARS-CoV-2 is a positive-sense, single-stranded, enveloped RNA virus with a genome of approximately 30 kb (Lu et al., 2020). The genome encodes for structural proteins, including the spike (S) protein, envelope (E) protein, membrane (M) protein, and nucleocapsid (N) protein, as well as non-structural proteins (NSPs) crucial for viral replication and immune evasion (Hu et al., 2021). The spike protein, responsible for receptor binding to the human angiotensin-converting enzyme 2 (ACE2) receptor (Wang et al., 2020), mediates viral entry into host cells and is a primary target for neutralizing antibodies and vaccine development (Walls et al., 2020). The virus replicates within the host cell cytoplasm, utilizing the host’s cellular machinery for viral RNA synthesis and protein production. The assembly of new virions occurs within the endoplasmic reticulum-Golgi intermediate compartment (ERGIC), followed by release through exocytosis (Calder et al., 2022).

The pandemic spurred intense research efforts aimed at understanding the virus’s biology, pathogenesis, and transmission dynamics, leading to the rapid development of diagnostic assays (Kevadiya et al., 2021; Liu et al., 2020), therapeutic interventions (Beigel et al., 2020; Gottlieb et al., 2022; Li et al., 2023; Toussi et al., 2023), and vaccines (Mattoo and Myoung, 2021; Patel et al., 2022). The development of mRNA vaccines (Baden et al., 2021; Corbett et al., 2020; Goel et al., 2021; Jackson et al., 2020; Polack et al., 2020; Thomas et al., 2021), in particular, represented a significant scientific achievement, providing high levels of protection against severe disease and reducing transmission rates (Corbett et al., 2020; Goel et al., 2021; Turner et al., 2021). However, the ongoing emergence of SARS-CoV-2 variants (Kim et al., 2023, 2024a; Raharinirina et al., 2025), characterized by increased transmissibility, immune evasion, and potential for increased disease severity, continues to pose challenges for pandemic control and necessitates ongoing surveillance and adaptation of public health strategies (Stowe et al., 2022). The long-term impact of the SARS-CoV-2 pandemic on global health and society remains a subject of intense investigation and concern.

Reverse genetics is now an indispensable tool for dissecting SARS-CoV-2 biology, offering unparalleled capabilities to investigate viral mechanisms, understand pathogenesis, and develop targeted interventions (Xie et al., 2021). The rapid advancement of these technologies was driven by the urgent need to decipher the novel virus, its replication cycle, and its interactions with the host immune system – questions that traditional forward genetics approaches could not address quickly or precisely enough (Amarilla et al., 2021; Xie et al., 2021). Reverse genetics enables researchers to introduce specific, defined mutations into the viral genome, allowing them to directly assess the resulting phenotypic changes and gain insight into the function of individual genes and regulatory elements (Fahnøe et al., 2022). This approach is particularly conducive to the study of viruses like SARS-CoV-2, where genetic manipulation can reveal significant contributions to virulence, transmission, and immune evasion. Moreover, efficient manipulation of viral genome through reverse genetics has led to the development of live attenuated (Liu et al., 2022; Nouailles et al., 2023; Schon et al., 2024; Suzuki Okutani et al., 2025; Trimpert et al., 2021; Ye et al., 2023) against SARS-CoV-2.

Several different reverse genetic systems for SARS-CoV-2 have been developed, each with its own advantages and limitations. These systems generally fall into two main categories: those based on in vitro ligation of cDNA fragments and those relying on yeast-based homologous recombination (Hou et al., 2020; Thi Nhu Thao et al., 2020; Wang et al., 2022). The in vitro ligation approach, exemplified by the use of infectious clones assembled from multiple cDNA fragments, offers precise control over the introduced mutations but can be technically challenging due to the large size and complexity of the SARS-CoV-2 genome. Yeast-based systems, on the other hand, simplify the assembly process by leveraging the homologous recombination machinery of yeast cells (Thi Nhu Thao et al., 2020). However, these systems may introduce unwanted mutations or rearrangements during the cloning process, requiring careful validation of the resulting viruses. Other strategies involve bacterial artificial chromosomes (BACs) or modified vaccinia Ankara (MVA) virus vectors to deliver and express the SARS-CoV-2 genome (García-Arriaza et al., 2021; Ye and Martinez-Sobrido, 2022). While BACs can maintain large DNA fragments stably, MVA vectors offer a safer platform for in vivo studies due to their restricted host range (Zhu et al., 2024). Despite their individual pros and cons, reverse genetics remain paramount in the continuous refinement of vaccines, antiviral therapeutics, and preventative strategies against SARS-CoV-2.

In this study, we introduce an innovative reverse genetics system for SARS-CoV-2 that employs a type IIS restriction enzyme to flank 40–50 nucleotide homology regions alongside fragmented genomes. This approach facilitates efficient and rapid sequence verification of viral genome fragments and the assembly of full-length cDNA through the Gibson assembly method in a BAC. Utilizing this system, we successfully generated recombinant viruses that express green fluorescent protein (GFP) or mNeonGreen (mNG) and assessed two distinct mammalian promoters to optimize reporter gene expression. This reverse genetics platform provides a robust tool for viral gene mutagenesis, enabling functional analyses of viral genes and laying the groundwork for the rational design of next-generation vaccines and therapeutics.

## Materials and Methods

### Cells

African green monkey kidney epithelial cells (Vero E6; ATCC, CRL-1586, USA) were purchased from the American Type Culture Collection (ATCC) (Chakraborty et al., 2024; Saha et al., 2024) and cultured in high-glucose Dulbecco’s modified Eagle’s medium (DMEM; Welgene, LM 001-02, Korea) supplemented with 10% fetal bovine serum (FBS; Welgene, S 001-07, Korea) and 1% Penicillin-Streptomycin (P/S; Welgene, LS 202-02, Korea) (Lee et al., 2024). Cells were maintained at 37℃ with 5% CO_2_ condition and sub-cultured every two day (Nam et al., 2024; Park et al., 2024).

### Assembly of a full-length SARS-CoV-2 cDNA

**Fig. 1.**
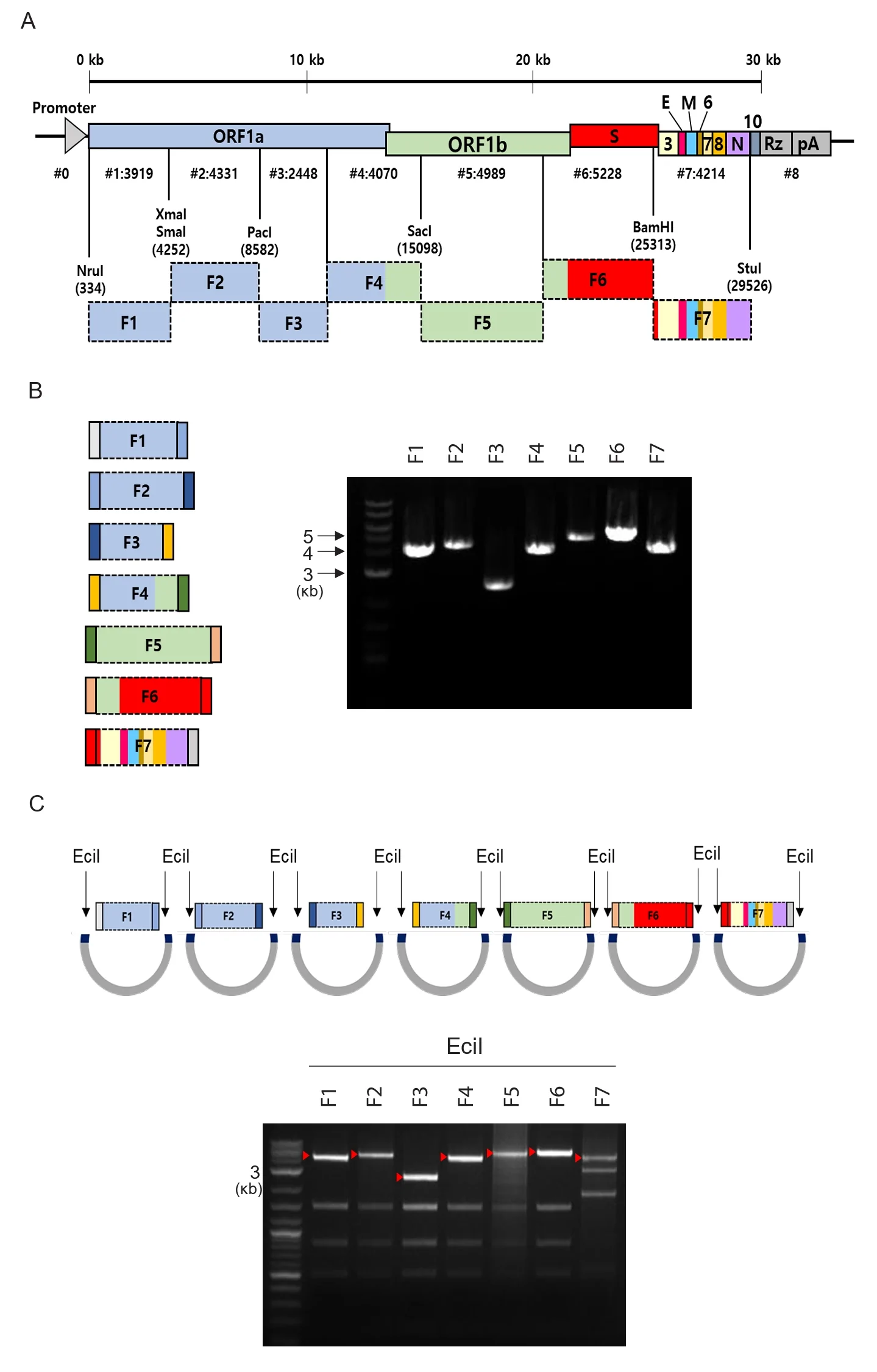
Reverse genetics of SARS-CoV-2. (A) Schematic of the synthetic fragment F08, which includes a promoter, 5′ UTR, 3′ UTR, hepatitis D virus Rz, and TKpA. The remainder of the SARS-CoV-2 genome was divided into seven fragments based on unique restriction sites (NruI, XmaI, PacI, SacI, BamHI, and StuI). Numbers next to the names of unique restriction enzymes indicate the genomic position of the first nucleotide in each enzyme’s recognition. Each fragment was synthesized from viral RNA using mouse moloney leukemia virus (MMLV) reverse transcriptase, and the resulting cDNA fragments were analyzed by electrophoresis on a 1% agarose gel. (B) Fragments 1–6 (F1–F6) were cloned into the pLPS-B vector, while Fragment 7 (F7) was inserted into the pMQ131 vector to ensure genetic stability. EciI (type IIS) restriction sites were introduced at both ends of each fragment to generate overhangs complementary to the homology regions of adjacent fragments. EciI digestion was performed to confirm correct insertion of all fragments into their respective vectors. (C) Fragments 1–7 were excised with EciI and visualized on a 1% agarose gel. Arrowheads indicate the position of each fragment.

A full-length cDNA clone of SARS-CoV-2 was constructed from a clinical isolate (NCCP43326) obtained from the National Culture Collection for Pathogens, with its reference sequence publicly available (NC_042212.2). To ensure the safety of personnel and the integrity of the research, all experiments using live SARS-CoV-2 viruses were performed in the biosafety level 3 facility at Jeonbuk National University, in accordance with the guidelines and regulations established by the Institutional Biosafety Committee. Fragment F0, which contains viral 5’ untranslated region (5′ UTR) to NruI site (position 334 nucleotide), and F8, which contains StuI (position 29526 nucleotide) to thymidine kinase polyadenylation signal sequence (TKpA), were synthesized de novo and cloned into the pMQ131 vector (Bionics, Korea) (Fig. 1). The elongation factor 1α/human T lymphotropic virus 1 long terminal repeat (EF1α/HTLV-1 LTR) promoter sequence was sourced from the pUNO1 vector (Invivogen, USA). Each fragment was amplified by polymerase chain reaction (PCR) of cDNA synthesized from total RNA extracted from virus-infected cells. The sequences of the primers used for PCR are provided in Table 1. The full-length SARS-CoV-2 cDNA was assembled from fragments 1 to 7 using the GeneArt^TM^ Gibson Assembly EX Master Mix (Thermo Fisher Scientific, USA) according to the manufacturer’s protocol. The final full-length cDNA plasmid was validated by next-generation sequencing (NGS; Macrogen, Korea) to ensure sequence fidelity. In addition, each genome fragment was individually cloned into shuttle vectors to allow flexible genetic manipulation and to minimize the risk of mutation accumulation during assembly steps. Fragments 1 to 6 were cloned into the pLPS-B blunt TOPO vector (Elpisbio, EBK-1002, Korea) using a blunt-end cloning strategy, following the manufacturer’s protocol. The fragment 7 was inserted into the pMQ131 vector by Sequence and Ligation-Independent Cloning (SLIC) (Bae et al., 2020; Islam et al., 2017; Jeong et al., 2012; Lee et al., 2020). For the SLIC reaction, T4 DNA polymerase (Enzynomics, DP004, Korea) was used to generate 3′ overhangs by incubating at 25°C for 2 min in the absence of dNTP to activate exonuclease activity, followed by inactivation with 100 mM EDTA. Primer sequences used for cloning are listed in Table 2.

To construct full-length cDNA containing either the hybrid EF1α-HTLV or CMV promoter, PCR amplification was performed using primers designed to introduce appropriate homology regions to each promoter sequence. The resulting promoter PCR products were inserted into the SgfI/NruI-digested full-length vector by Gibson Assembly. The primer sequences used for this step are provided in Table 3.

A reporter gene expression cassette encoding GFP or mNG was inserted downstream of the nucleocapsid (N) gene, regulated by the ORF7a transcription regulatory sequence (TRS). The pMQ131-nCoV-08 plasmid was linearized using StuI restriction enzyme, and PCR-amplified fluorescent protein coding sequences flanked by homology arms were integrated by SLIC. Primer sequences used for amplification are provided in Table 4.

### RNA extraction and synthesis cDNA

Viral RNA was extracted using the QIAamp Viral RNA Mini Kit (Qiagen, 52904, USA) according to the manufacturer’s instructions (Cho et al., 2024; Kim et al., 2024b; Moon et al., 2024). Complementary DNA (cDNA) was synthesized from the extracted RNA using M-MLV Reverse Transcriptase (Promega, M170A, USA). Seven cDNA fragments were generated, corresponding to regions amplified by the primers listed in Table 1. The primers were designed to produce overlapping fragments (25–40 bp) to facilitate seamless assembly during downstream applications.

### Virus rescue and quantification

Vero E6 cells were seeded in 6-well plates at a density of 2.5 × 10⁵ cells/well and incubated overnight at 37°C. On the following day, 2 µg of infectious clone DNA was diluted in 100 µl of Opti-MEM I Reduced Serum Medium (Gibco, 31985-070, USA). In parallel, 4 µl of TransIT-LT1 transfection reagent (Mirus, MIR2304, USA) was diluted in 100 µl of Opti-MEM and incubated at room temperature for 5 min. The DNA and transfection reagent solutions were then combined, incubated for an additional 15 min at room temperature, and subsequently added dropwise to the cells. Supernatants were harvested as P0 virus stocks 72–96 h post-transfection or upon observation of 50% cytopathic effect (CPE). All experiments involving live virus were performed in a Biosafety Level 3 (BSL-3) facility at the Korea Zoonosis Research Institute, Jeonbuk National University.

For quantification of viruses, plaque assays were employed (Lee et al., 2023; Nie et al., 2023). Briefly, Vero E6 cells were seeded at 3.7 × 10⁵ cells/well in 6-well plates and incubated overnight at 37°C. Serially diluted virus samples (500 µl) were used to infect cells for 1  h at 37°C with gentle rocking every 20 min. Post-infection, a 1% HiQ agarose overlay containing 2% FBS, 1% penicillin-streptomycin, and 1× DMEM was applied (3 ml/well) and solidified at room temperature. Plates were incubated at 37°C for 2–3 days until plaques formed. Cells were fixed with 4% paraformaldehyde (PFA), and plaques were visualized by crystal violet staining after agarose removal. The size of viral plaques was measured using ImageJ software (NIH, USA). Quantification of viral RNA was performed as previously described (Kim et al., 2023, 2024a). Briefly, total RNA was extracted using GENTi^TM^ 32 Advanced system (Precision System Science, Japan), and qRT-PCR was conducted with DiaStar^TM^ OneStep Multiplex qRT-PCR Kit (Solgent, SRQ11-K, Korea), targeting the N gene of SARS-CoV-2. The following primers and probe were used:

Forward: 5′-GACCCCAAATCAGCGAAAT-3′

Reverse: 5′-TCTGGTTACTGCCAGTTGAA-3′

Probe: 5′-FAM-GACCCCAAATCAGCGAAAT-TAMRA-3′

### Flow cytometry

Vero E6 cells were infected with the recombinant virus at a multiplicity of infection (MOI) of 0.1. At 24 h post-infection (hpi), cells were harvested by trypsinization and subsequently fixed in 4% PFA for 10 min at room temperature. Following fixation, cells were washed twice with FACS buffer (phosphate-buffered saline supplemented with 2% FBS) and resuspended in 200 μl of the same buffer. Flow cytometric analysis was conducted using the BD FACSLyric^TM^ system (BD Biosciences, USA). Single, viable cells were identified and gated based on forward scatter (FSC) and side scatter (SSC) parameters. GFP-expressing cells were detected and gated using FITC fluorescence. The mean fluorescence intensity (MFI) of the gated population was subsequently quantified.

### RNA structure prediction

The secondary structures of the SARS-CoV-2 5′ UTR and a chimeric HTLV-1 5′ LTR-SARS-CoV-2 5′UTR RNA were predicted using the MFold web server (Zuker, 2003). FASTA sequences were analyzed assuming circular RNA, visualizing base pairs with line representations, and imposing a flat exterior loop type.

### Statistical analysis

Statistical analyses were conducted using the Student’s *t*-test. A p-value of less than 0.05 was considered statistically significant (*p* < 0.05: *, *p* < 0.01: **, *p* < 0.001: ***, *p* ≥ 0.05: ns). Data are expressed as mean ± standard deviation (SD).

## Results

### A modular reverse genetic system for SARS-CoV-2 was established using Gibson assembly

To construct a full-length cDNA clone of SARS-CoV-2, which has an approximate genome size of 30 kb, the viral genome was divided into seven segments. Each segment was assigned based on the positions of unique restriction enzyme sites present in the viral genome, facilitating the efficient generation of a full-length infectious clone both by conventional cut-and-paste cloning and Gibson assembly. The viral genome, as depicted in Fig. 1A, is flanked at the 3’ end by a ribozyme (Rz) and a thymidine kinase polyadenylation signal sequence (pA). This configuration facilitates the precise synthesis of the viral RNA genome following the transfection of cDNA into mammalian cells. Each genome fragment (Fig. 1A and 1B) was amplified from cDNA prepared from the Korean clinical isolate obtained from the National Culture Collection for Pathogens (NCCP 43326), with the sequence published under the accession number NC_045512.2 in PubMed. Fragments 1 through 7 were engineered to include a type IIS restriction enzyme site (EciI) and homology regions corresponding to adjacent viral genome fragments. This design facilitates the generation of sequence-verified fragments, which serve as building blocks for the assembly of a full-length cDNA using Gibson assembly (Fig. 1B), allowing the integrity and modularity of the full-length cDNA cloning of the viral genome (Fig. 1C). Furthermore, the system heightens the accessibility of the SARS-CoV-2 RNA genome for molecular biological manipulation, enabling efficient construction of genetically modified viral cDNA clones when desired.

### Recombinant SARS-CoV-2 was successfully rescued, exhibiting uniform plaque sizes in contrast to those observed in a clinical isolate while demonstrating comparable growth kinetics

**Fig. 2.**
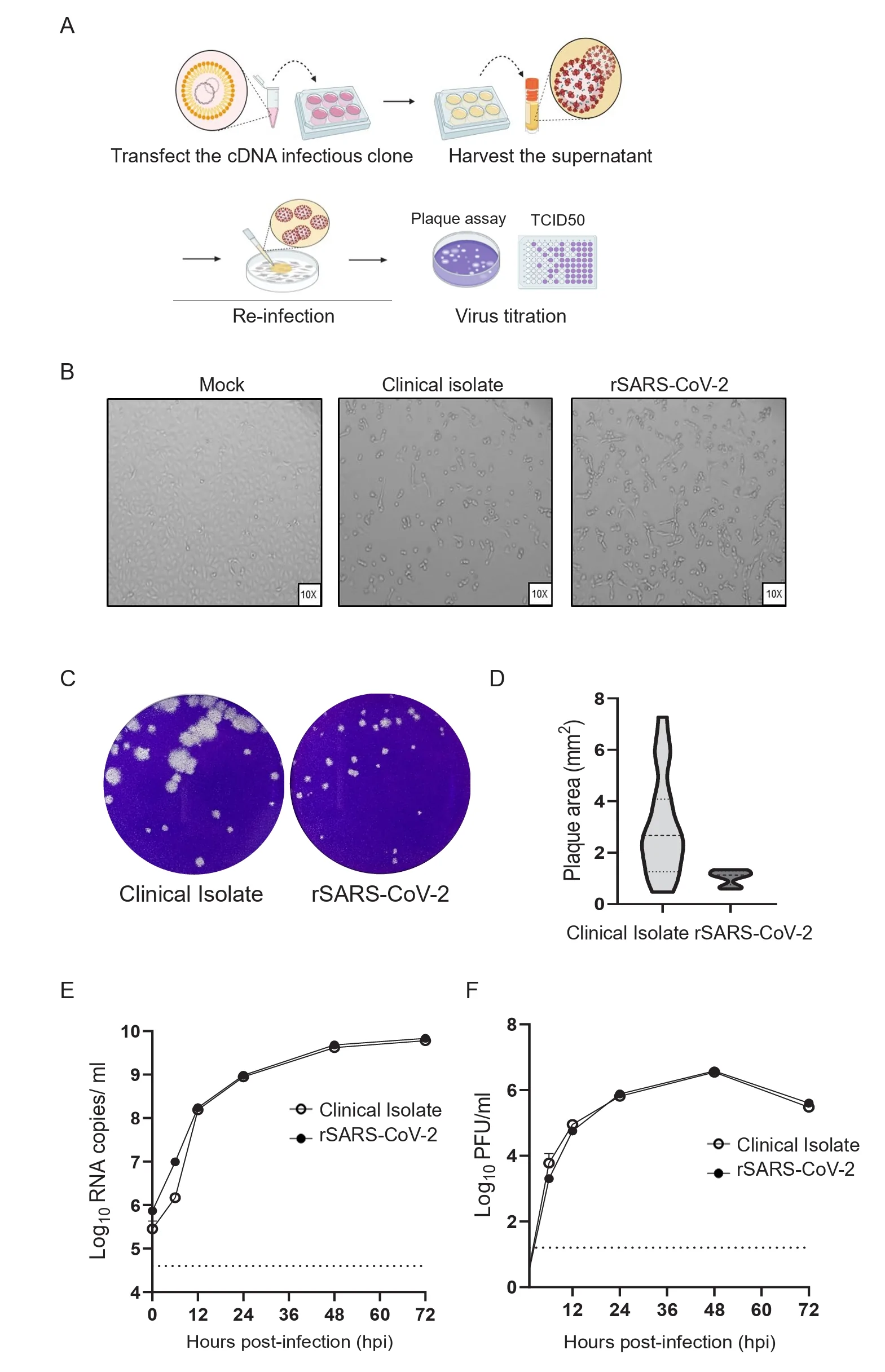
Characterization of recombinant SARS-CoV-2 production and replication. (A) Schematic of recombinant virus production: Vero E6 cells were transfected with 2 µg of the SARS-CoV-2 infectious clone. Supernatants were harvested upon CPE observation (B) and titrated via TCID_50_ or plaque assay. (C) Plaque morphology comparison between clinical isolate (WT) and recombinant virus. (D) Quantification of plaque sizes (mean ± SD) using ImageJ (n = 50 plaques per group; ^***^p < 0.001, unpaired t-test). Growth kinetics in Vero E6 cells (MOI = 0.1): viral RNA copies (qRT-PCR, E) and infectious titers (plaque assay, F) were measured at 0, 12, 24, 36, 48, and 72 h post-infection (hpi).

To rescue a recombinant SARS-CoV-2 (rSARS-CoV-2), Vero E6 cells were transfected with 2 µg of plasmid DNA using Mirus LT-1 transfection reagent in a 6-well plate (Fig. 2A). Two to three days post-transfection, CPE was evident in the transfected cell monolayer, demonstrating the presence of replication-competent recombinant viruses. These viruses were subsequently harvested to create a P0 stock, which was then passaged and amplified two to three times in the same cell line to generate a virus stock. Infection of Vero E6 cells with rSARS-CoV-2, in conjunction with a clinical isolate, exhibited comparable levels of CPE at 24 h post-infection, as illustrated in Fig. 2B, indicating a similar replication capability between the two viruses. The resulting virus stocks were titrated by both the plaque assay (Fig. 2C) and the TCID50 method (data not shown). Notably, the titers obtained from the plaque assay were approximately 1.5-fold higher than those measured by the TCID50 method, indicating a greater sensitivity of the plaque assay in quantifying infectious viral particles. Accordingly, subsequent experiments utilized plaque assays to quantify the titers of infectious virus present in each sample.

Of note, plaques formed by the clinical isolate were heterogeneous in both size and morphology, whereas those produced by the rSARS-CoV-2 were uniform and consistent (Fig. 2C and 2D). This observation indicates that virus stocks generated through reverse genetics exhibit greater homogeneity compared to clinical isolates, which often display heterogeneity due to multiple passages. These findings highlight the value of the reverse genetics platform as a standardized tool for SARS-CoV-2 research, enhancing experimental reproducibility.

To assess the replicative competency of rSARS-CoV-2, Vero E6 cells were infected at a MOI of 0.1. Supernatants were collected at 6, 12, 24, 48, and 72 h post-infection, and viral RNA was quantified following extraction. Quantitative RT-PCR analysis showed no significant differences in viral RNA copy numbers between the recombinant and clinical isolate strains at any time point (Fig. 2E). In parallel, plaque assays performed on the same supernatants demonstrated comparable infectious virus titers between the two strains (Fig. 2F). Together, these results indicate that the recombinant virus exhibits replication kinetics equivalent to those of the clinical isolate, supporting its utility as a reliable and standardized model for SARS-CoV-2 research.

### The GFP-expressing rSARS-CoV-2 was successfully rescued and exhibited growth kinetics comparable to those of the non-fluorescent rSARS-CoV-2

**Fig. 3.**
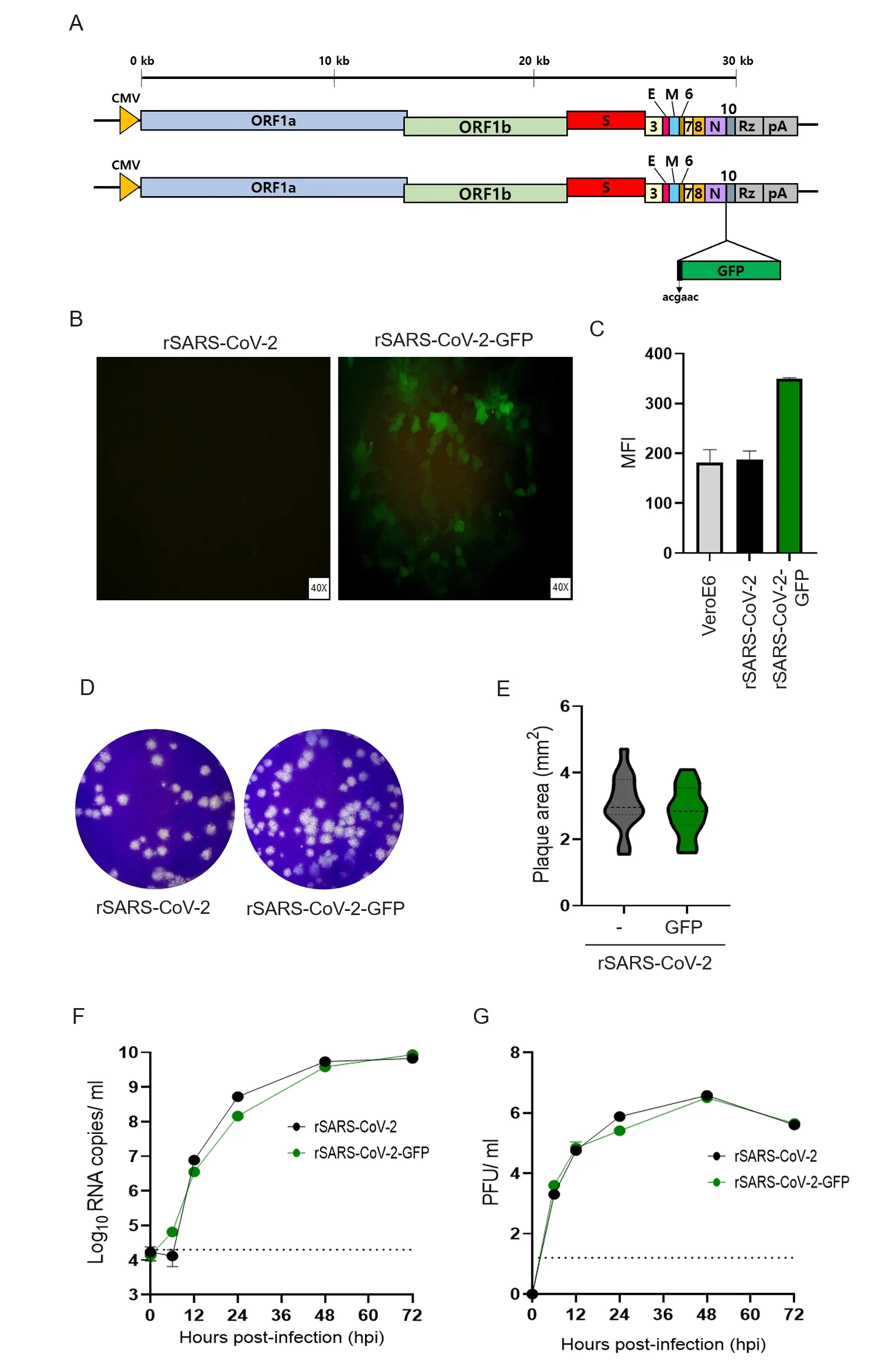
Characterization of rSARS-CoV-2 expressing GFP downstream of the N gene under the control of the ORF7a. (A) GFP was inserted downstream of the N gene and placed under the control of the ORF7a TRS to enable its expression. (B) Vero E6 cells were infected at a MOI of 0.1, and GFP expression was visualized by fluorescence microscopy at 1 day post-infection (dpi). (C) GFP fluorescence intensity was quantified by flow cytometry and reported as MFI. (D) Supernatants from infected Vero E6 cells (MOI 0.1, 1 dpi) were collected for plaque assay. (E) Plaque sizes were measured and analyzed using ImageJ software. (E) Viral replication kinetics were assessed by harvesting samples at 6, 12, 24, 36, 48, and 72 h post-infection, followed by quantification of viral RNA copy number (F) and plaque-forming units (PFU) (G).

To enable real-time visualization and expand the applicability of rSARS-CoV-2, we constructed a virus expressing a fluorescent reporter gene. The GFP gene was inserted at the 3′ end of the viral genome, downstream of the TRS for ORF7a, a region known to drive strong downstream gene expression (Fig. 3A), thereby minimizing disruption to endogenous viral gene function. Successful GFP expression in infected cells was confirmed by fluorescence microscopy (Fig. 3B), and flow cytometry demonstrated a significant increase in MFI in cells infected with the GFP-expressing virus compared to control virus (Fig. 3C). Plaque assays indicated that GFP insertion did not alter plaque size or morphology relative to the non-GFP recombinant virus (Fig. 3D and 3E). To assess the impact of GFP expression on viral replication, Vero E6 cells were infected at a MOI of 0.1, and viral supernatants were collected at various time points post-infection (6–72 hpi). Quantitative RT-PCR targeting the N gene revealed no significant differences in viral RNA copy numbers between GFP-expressing and non-expressing viruses (Fig. 3F). Consistently, plaque assays performed on these samples showed comparable infectious viral titers in both groups (Fig. 3G). Together, these findings indicate that GFP incorporation does not affect viral replication kinetics, supporting the utility of fluorescent rSARS-CoV-2 for applications such as functional genomics, virus neutralization assay and antiviral drug screening.

### A rSARS-CoV expressing mNG exhibited enhanced fluorescence

**Fig. 4.**
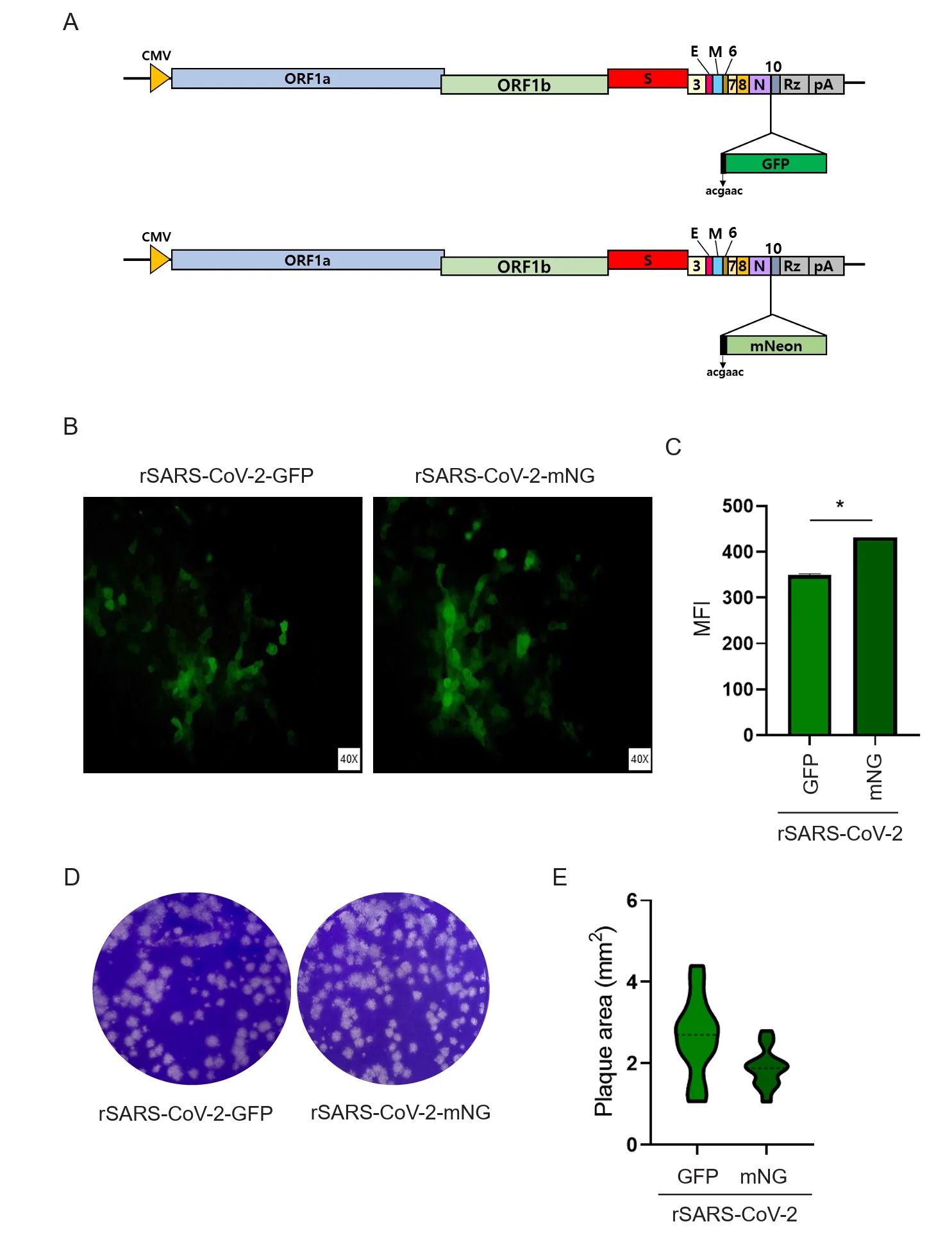
Generation and characterization of a mNG-expressing recombinant virus. (A) A recombinant virus was constructed by substituting the GFP gene with the mNG gene. (B) Fluorescence microscopy images show the expression of GFP and mNG in infected Vero E6 cells. (C) Fluorescence intensity of GFP and mNG was quantified in infected cells by flow cytometry, and MFI values were compared. (D) Plaque assays were performed by serially diluting viral stocks and infecting Vero E6 cells to evaluate plaque formation. (E) Plaque sizes produced by the recombinant viruses were measured and analyzed using ImageJ software. Statistical significance is indicated as follows: ns, *p* ≥ 0.05; ^*^, *p* < 0.05; ^**^, *p* < 0.01; ^***^, *p* < 0.001.

To improve the brightness and maturation efficiency of fluorescent signals in recombinant virus systems, we engineered a novel recombinant virus expressing mNG, a next-generation green fluorescent protein distinguished by its superior photophysical properties, including greater brightness and faster chromophore maturation, compared to conventional GFP (Shaner et al., 2013). The recombinant virus was generated by substituting the GFP gene in the parental construct with the mNG coding sequence, while keeping all other viral components unchanged to ensure comparability between constructs (Fig. 4A). Fluorescence microscopy revealed that both GFP- and mNG-expressing viruses produced robust and consistent fluorescence in infected cells (Fig. 4B). Quantitative analysis demonstrated that the mNG-expressing virus exhibited approximately 1.2-fold higher fluorescence intensity than its GFP-expressing counterpart, a difference that was statistically significant (p = 0.037), indicating that replacing GFP with mNG leads to a measurable enhancement in fluorescent signal output (Fig. 4C). Plaque assays assessing viral replication and cytopathic effect showed that both recombinant viruses formed homogeneous plaques of similar size (Fig. 4D and 4E), suggesting that incorporation of mNG does not adversely affect viral propagation or plaque-forming efficiency. Collectively, these results establish mNG as a superior alternative to GFP for recombinant virus systems, offering enhanced fluorescence performance without compromising viral fitness.

### Recombinant viruses generated using the CMV promoter displayed more robust plaque formation relative to those driven by the EF1α/HTLV promoter

**Fig. 5.**
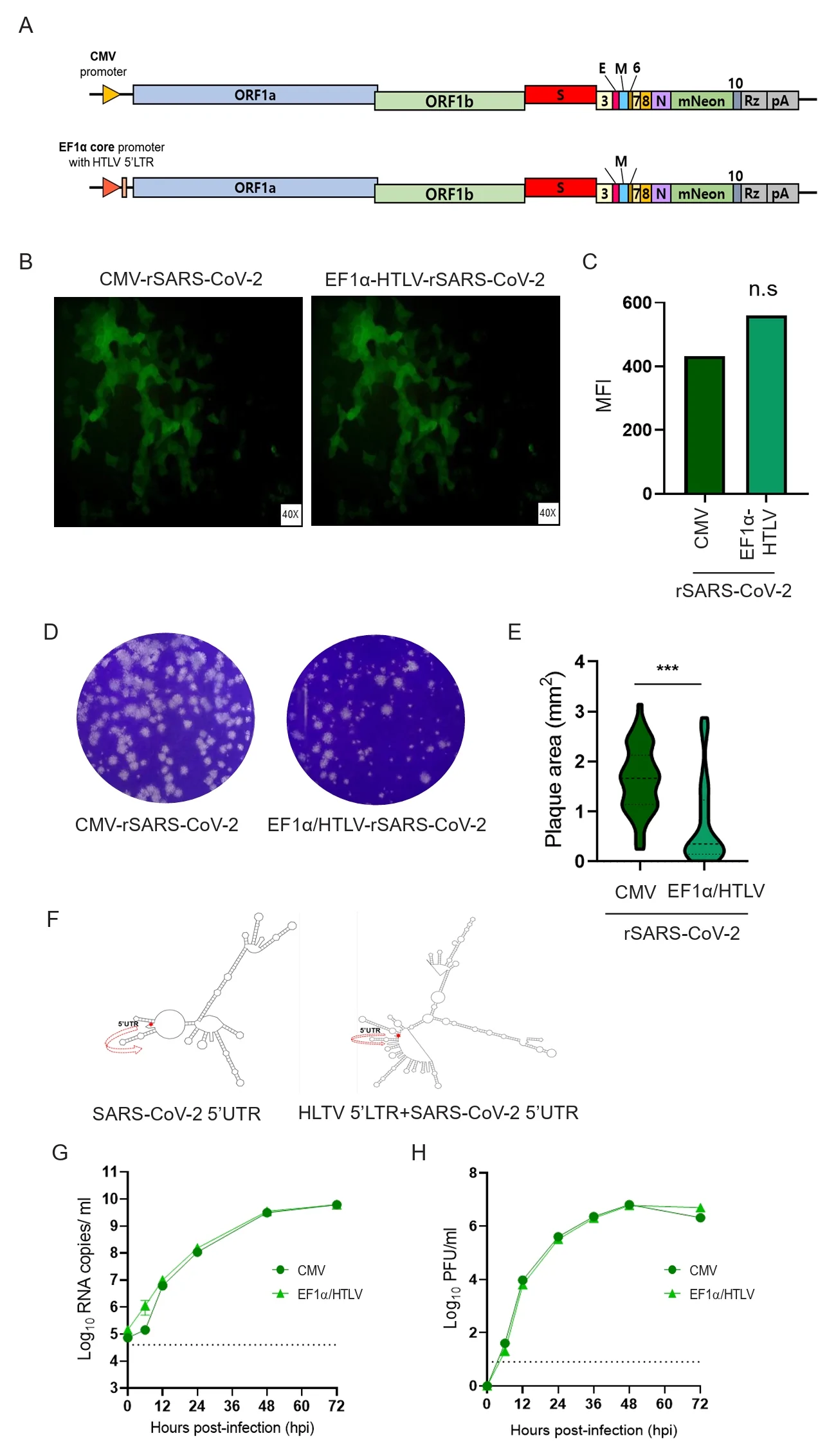
Schematic and functional characterization of cDNA infectious clones driven by CMV and EF1α/HTLV promoters. (A) Schematic of cDNA infectious clones driven by CMV and EF1α/HTLV promoters (B) mNG expression visualized by fluorescence microscopy in Vero E6 cells infected with recombinant viruses under the control of either the CMV or EF1α/HTLV promoter. (C) Quantification of mNG expression by MFI via flow cytometry. (D) Assessment of viral infectivity: Vero E6 cells were infected with each recombinant virus at an MOI of 0.1, and plaque morpology was assesed by plaque assay. (E) Plaque size of each recombinant virus was analyzed using ImageJ software. (F) RNA secondary structure prediction of the SARS-CoV-2 5'UTR and a hybrid 5'UTR containing the HTLV-1 5'LTR was made using the MFold tool. Growth kinetics of recombinant viruses in Vero E6 cells infected at an MOI of 0.1, with supernatant harvested at indicated time points (0, 6, 12, 18, 24, 48, and 72 h post-infection) and viral replication assessed by RNA copy number (G) and PFU quantification (H). Statistical significance: ns, p ≥ 0.05; ^*^, p < 0.05; ^**^, p < 0.01; ^***^, p < 0.001.

To assess whether a strong promoter enhances the fluorescence intensity of mNG-expressing rSARS-CoV-2, we employed a hybrid promoter consisting of the human EF1α promoter and the HTLV 5’ LTR, which is derived from the pUNO1 plasmid (InvivoGen) and known strongly to induce expression of downstream gene (Fig. 5A). Two recombinant viruses were constructed, each expressing mNG under the control of either the CMV promoter or the EF1α-HTLV hybrid promoter, to evaluate the effects of promoter elements on gene expression and viral replication (Fig. 5A). Fluorescence microscopy confirmed robust mNG expression in cells infected with both recombinant viruses. Quantitative analysis indicated comparable fluorescence intensity between the two recombinant viruses (Fig. 5B and 5C). Despite similar levels of fluorescence, notable differences in plaque morphology were observed, with the hybrid promoter-driven virus consistently producing smaller plaques than the CMV promoter-driven virus (Fig. 5D and 5E). To explore the basis for these phenotypic differences, the RNA secondary structures of the viral 5’UTRs was predicted using the MFold in the presence of either the CMV or the hybrid promoter, displaying quite large differences (Fig. 5F). To evaluate the replication capacity of the two recombinant viruses, viral growth kinetics were analyzed at a MOI of 0.1. As shown in Fig. 5G and 5H, no significant differences in viral titers were observed between the two recombinant viruses throughout the course of infection. Taken together, these findings indicate that while both promoters effectively drive robust reporter expression, the CMV promoter may be more advantageous for generating reporter-expressing rSARS-CoV-2.

## Discussion

The establishment of reverse genetics systems for SARS-CoV-2 has been crucial in advancing our understanding of viral replication, pathogenesis, and antiviral development (Fahnøe et al., 2022; Hou et al., 2020; Wang et al., 2022; Xie et al., 2021). A key step in this process involves creating a full-length cDNA clone of the viral genome (Figs. 1 and 2), which can then be manipulated to introduce specific mutations or reporter genes (Figs. 3 and 4). The initial creation of a reverse genetics system for SARS-CoV-2 involved segmenting the viral genome into smaller fragments that can be assembled using Gibson assembly (Fig. 1). This approach allows for efficient manipulation of the viral genome and reconstruction of genetically modified viral cDNA clones. The ability to generate recombinant viruses from cDNA clones has been a significant breakthrough, enabling researchers to study the virus in a controlled and reproducible manner. The proposed methods offer several advantages over previously published approaches: (1) Each fragment is individually maintained in a plasmid, eliminating the need for repeated PCR amplification or de novo synthesis; (2) Fragments can be excised from their plasmids using a Type IIs restriction enzyme, generating products with precise flanking homology regions for Gibson assembly and thereby reducing the need for repeated sequence validation; and (3) For mutagenesis, mutations are introduced and sequence-verified in individual fragments, which can then be efficiently exchanged with their corresponding wild-type counterparts. Overall, this method provides a more convenient workflow and higher efficiency compared to existing techniques.

The first critical validation step is ensuring that the recombinant virus replicates with comparable efficiency to clinical isolates (Figs. 2, 3, and 5). Studies have demonstrated that rSARS-CoV-2 exhibits similar replication kinetics to its clinical counterparts, validating its use as a reliable model for research (Fig. 2). rSARS-CoV-2 generated using reverse genetics exhibited markedly more homogeneous plaque morphology compared to clinical isolates (Fig. 2C, 3D, 4D, and 5D), reflecting the genetic uniformity inherent to reverse genetics-derived viral populations (Chiem et al., 2021; Xie et al., 2021). This homogeneity is a valuable attribute, as it enhances the reproducibility of experimental outcomes in both in vitro and in vivo pathogenesis studies, facilitating more consistent and interpretable results (Xie et al., 2021; Ye et al., 2020). Furthermore, the introduction of fluorescent reporter genes, such as GFP or mNG, into the viral genome allows for real-time monitoring of viral infection and replication (Figs. 3 and 4). These fluorescently labeled viruses have broad applications in functional genomics and antiviral drug screening, offering a powerful tool for studying virus-host interactions and evaluating the efficacy of potential therapeutics (Hou et al., 2020; Thi Nhu Thao et al., 2020).

Efforts to optimize recombinant virus systems have focused on enhancing the brightness and stability of fluorescent signals (Chiem et al., 2021; Hou et al., 2020; Ye et al., 2021). The use of mNG has been shown to provide a brighter and more stable signal compared to traditional GFP (Fig. 4). Additionally, the choice of promoter driving the expression of the fluorescent reporter gene can impact plaque formation, with the CMV promoter resulting in more robust plaques compared to the EF1α/HTLV promoter (Fig. 5). The rSARS-CoV-2 generated using the hybrid promoter exhibited viral growth kinetics comparable to those driven by the CMV promoter (Fig. 5F and 5G). Notably, however, the plaque size of the hybrid promoter-driven rSARS-CoV-2 was substantially smaller and heterogenous than that observed with the CMV promoter-driven virus (Fig. 5D and 5E). Smaller plaque sizes observed in our study may indicate attenuated viral replication, potentially attributable to alterations in RNA structure as predicted (Fig. 5F). Notably, pronounced differences in the predicted conformations of the viral 5′ UTR suggest that the highly structured HTLV-LTR element could modulate the architecture of the viral 5′ UTR, thereby influencing RNA translation efficiency and, in turn, impacting viral replication and plaque formation (Nakano and Watanabe, 2012, 2022). The regulatory complexity of the 5′ UTR and LTR regions is well-documented in retroviruses, where such structural elements and their interactions with viral and host factors can significantly affect gene expression and replication dynamics (Nakano and Watanabe, 2012, 2022). These findings highlight the importance of carefully considering the design and components of recombinant virus systems to maximize their utility in research applications.

Taken altogether, we present an efficient and robust reverse genetics system for SARS-CoV-2 that enables the convenient and rapid generation of mutant viruses carrying specific genetic modifications. This system streamlines the introduction of targeted mutations into the viral genome, thereby facilitating rational vaccine design and high-throughput antiviral drug screening.

## Figures and Tables

**Table 1.** Primer information for cDNA synthesis of each fragment

Primer information		
	PCR Primer sequence	
	Forward primer	Reverse primer
F1	5'-cacacgtccaactcagtttgcctgttttacaggttcgcga-3'	5'-ccatttaaaccctgacccgggtaagtg-3'
F2	5'-cagacaattatataaccacttacccgggtc-3'	5'-ggaacacaagtgtaactttaattaactgcttc-3'
F3	5'-gttaataattggttgaagcagttaattaaagttacacttgtgttcc-3'	5'-ggactaaaactaaaagtgaagtcaaaattgtgag-3'
F4	5'-gttactcacaattttgacttcacttttagttttagtcc-3'	5'-caccagctacggtgcgagctctattc-3'
F5	5'-cattagtgcaaagaatagagctcgcacc-3'	5'-ccattaagactagcttgtttgggacctacag-3'
F6	5'-ggtttacaaccatctgtaggtcccaaacaag-3'	5'-gtcttcatcaaatttgcagcaggatccac-3'
F7	5'-ctcaagggctgttgttcttgtggatcc-3'	5'-cgtttatatagcccatctgccttgtgtgg-3'
		
		

**Table 2.** Primer information for single-fragment cloning using the SLIC method

Primer information		
	PCR Primer sequence	
	Forward primer	Reverse primer
F1	5'-ggatccGGCGGAtaccagtgtgtttgcctgttttacaggttcgcgacgtgctcgtac-3'	5'-ctcgagGGCGGAtaccagtgtccatttaaaccctgacccgggtaagtggttatataattgtc-3'
F2	5'-ggatccGGCGGAtaccagtgtttatataaccacttacccgggtcagggtttaaatg-3'	5'-ctcgagGGCGGAtaccagtgtcacaagtgtaactttaattaactgcttcaaccaattattaacaattttacc-3'
F3	5'-ggatccGGCGGAtaccagtgtaattggttgaagcagttaattaaagttacacttgtgttcctttttg-3'	5'-ctcgagGGCGGAtaccagtgtctaaaactaaaagtgaagtcaaaattgtgagtaacaaccag-3'
F4	5'-ggatccGGCGGAtaccagtgtgttactcacaattttgacttcacttttagttttagtccagagtac-3'	5'-ctcgagGGCGGAtaccagtgtcaccagctacggtgcgagctctattctttgcactaatggc-3'
F5	5'-ggatccGGCGGAtaccagtgtttagtgcaaagaatagagctcgcaccgtagctg-3'	5'-ctcgagGGCGGAtaccagtgtaagactagcttgtttgggacctacagatggttgtaaacc-3'
F6	5'-tctagaGGCGGAtaccagtgtttacaaccatctgtaggtcccaaacaagctagtcttaatg-3'	5'-ctcgagGGCGGAtaccagtgtatcaaatttgcagcaggatccacaagaacaacagccc-3'
F7	5'-cctgcaggGGCGGAtaccagtgtggctgttgttcttgtggatcctgctgcaaatttgatgaaga-3'	5'-gggcccGGCGGAtaccagtgtggtctgcatgagtttaggcctgagttgagtcagcac-3'

**Table 3.** Primer sequences for promoter substitution

Primer information	
	PCR Primer sequence
CMVp-nCoV-0-vec-linear-F	5'- ccatgagcagtgctgactcaactcaggcctaaactcatgcagaccac -3'
CMVp-nCoV-0-vec-linear-R	5'- gtctccaaagccacgtacgagcacgtcgcgaacctgtaaaacaggcaaac -3'
CMVp-Gibson-F	5'- ggtccgggccattatggccacctggtggatctgcgatcgcgacattgattattgactagttattaatagtaatc -3'
CMVp-Gibson-R	5'- gttggttggtttgttacctgggaaggtataaacctttaatagctctgcttatatagacctccc -3'
nCoV-0-Gibson-F	5'- attaaaggtttataccttcccaggtaacaaac -3'
nCoV-0-Gibson-R	5'- cttaattaagcgcgccccggggcgcgctcgcgaacctgtaaaacaggc -3'

**Table 4.** Primers for cloning of fluorescent reporter genes

Primer information	
	PCR Primer sequence
pMQ131-CMV-vec-linear-F	5'- ggaggctaactgaaacacggaa -3'
pMQ131-CMV-vec-linear-R	5'- agctctgcttatatagacctcccac -3'
SLIC-7a-TRS-GFP-F	5'- tgctgactcaactcaggcctaaacgaacatggtgagcaagggcgag -3'
SLIC-7a-TRS-mNeonGreen-F	5'- tgctgactcaactcaggcctaaacgaacatggtgagcaagggcgag -3'
SLIC-7a-TRS-GFP or mNeonGreen-R	5'- ccagtgtggtctgcatgagttt -3'
